# Assessment of Platelet and Plasma Serotonin in Canine Pulmonary Hypertension Secondary to Degenerative Mitral Valve Disease

**DOI:** 10.3389/fvets.2021.695492

**Published:** 2021-06-15

**Authors:** Nattawan Tangmahakul, Pussadee Makoom, Sirilak Disatian Surachetpong

**Affiliations:** ^1^Department of Veterinary Medicine, Faculty of Veterinary Science, Chulalongkorn University, Bangkok, Thailand; ^2^Veterinary Diagnostic Laboratory, Faculty of Veterinary Science, Chulalongkorn University, Bangkok, Thailand

**Keywords:** canine, degenerative mitral valve disease, pulmonary hypertension, plasma, platelet, serotonin

## Abstract

**Background:** Pulmonary hypertension (PH) is a common complication of degenerative mitral valve disease (DMVD), the most common cardiovascular disease in dogs. Serotonin has been suspected to play a role in the pathogenesis of PH, so this study aimed to investigate the differences in platelet and plasma serotonin between normal, DMVD and DMVD with PH (DMVD+PH) dogs.

**Materials and Methods:** Sixty-two small-breed dogs were enrolled to the study and divided into the normal (*n* = 22), DMVD (*n* = 20), and DMVD+PH (*n* = 20) groups. The platelet and plasma serotonin concentrations were measured by the competitive ELISA.

**Results:** The Kruskal–Wallis revealed the difference among the four groups of normal (179.73 [102.37–352.24] ng/10^9^ platelets), DMVD (325.99 [96.84–407.66] ng/10^9^ platelets), DMVD with intermediate probability of PH (291.11 [106.69–400.84] ng/10^9^ platelets) and DMVD with high probability of PH (35.82 [2.69–126.35] ng/10^9^ platelets) (*p* = 0.014). The Dunn's *post-hoc* test showed a decrease in the platelet serotonin concentration of the DMVD dogs with high probability of PH compared to the DMVD group (*p* = 0.008). The plasma serotonin concentration was not different between normal, DMVD, and DMVD+PH dogs.

**Conclusion:** In conclusion, a decrease in platelet serotonin concentration, which is associated with a degree of PH probability was found in DMVD dogs with PH. Further studies investigating roles of platelet serotonin in PH secondary to DMVD should be performed.

## Introduction

Pulmonary hypertension (PH) is an abnormal elevation of pulmonary vascular pressure. PH in dogs is classified into six groups according to the American College of Veterinary Internal Medicine (ACVIM) consensus statement guidelines (1). The classification group of PH includes pulmonary arterial hypertension (PAH), PH due to left heart disease, PH secondary to respiratory disease and/or hypoxia, pulmonary emboli/thrombi/thromboemboli, parasite disease, and PH with multifactorial or unclear mechanisms (1). PH is commonly found as a complication of degenerative mitral disease (DMVD), the most common cardiac disease in older small-breed dogs (2). The clinical signs of PH are not specific and depend on the disease severity (1, 3). Coughing, changes in breathing sound and pattern, exercise intolerance, respiratory distress, heart murmurs located in the tricuspid area, cyanosis, and syncope can be found in dogs with PH (3–7). Echocardiography is used for the diagnosis of PH in dogs by measuring the tricuspid regurgitation jet velocity and calculating for the estimated pulmonary arterial pressure (PAP). Echocardiographic signs of PH involving right ventricle, pulmonary artery, right atrium, and caudal vena cava and the estimated PAP assessed by spectral Doppler echocardiography have been evaluated for staging the probability of PH (1). Several studies have reported the association of circulating serotonin in humans with PH (8–10). Involvement of serotonin in PH has been reported because PH has been found increasingly in women treated with some anorectic drugs, which are serotonin transporter (SERT) substrates that can increase extracellular serotonin concentration (11).

Serotonin is a biogenic amine involved in the physiology, development, and dysfunction of several organs, including the cardiovascular system (12). Serotonin is produced mainly by the enterochromaffin cells of the intestine, and 99% of circulating serotonin is taken up and stored in dense granules of platelets (13, 14). A previous study investigating serotonin in platelets, plasma, left ventricular myocardial tissues, and mitral valve leaflets of dogs revealed that dogs with DMVD had increased platelet serotonin levels together with higher serotonin levels in mitral valves and left ventricular myocardial tissues suggesting that serotonin in platelets may play a role in pathogenesis of DMVD (15). Serotonin is synthesized from tryptophan through a pathway involving the enzyme, tryptophan hydroxylase-1 (TPH-1) in pulmonary artery endothelial cells (16). It is then transferred to pulmonary artery smooth muscle cells via SERT and serotonin receptors to mediate the downstream effectors in the serotonin signaling pathway, causing contraction, proliferation and differentiation of pulmonary arterial walls (17–20). Pulmonary arterial wall thickening has been found with overexpression of proteins related to the serotonin signaling pathway, including TPH-1, SERT, serotonin receptor 2A, extracellular signal-regulated kinase 1/2 (ERK1/2) and phosphorylated ERK1/2 (pERK1/2) in dogs with PH secondary to DMVD (21). However, the association of circulating serotonin and PH secondary to DMVD in dogs has not been studied. We hypothesized that circulating serotonin concentrations in normal dogs and in DMVD dogs with and without PH are different. The present study aimed to investigate the difference in platelet and plasma serotonin concentrations in normal dogs and DMVD dogs with and without PH.

## Materials and Methods

### Animals

The protocol used in this study was approved by the Institutional Animal Care and Use Committee, Faculty of Veterinary Science, Chulalongkorn University (number 1831053). The client-owned dogs enrolled in the study were visited at the Small Animal Veterinary Teaching Hospital, Faculty of Veterinary Science, Chulalongkorn University, Thailand. The dogs included in the study were small-breed dogs aged 7–15 years old and weight <10 kg. Their history and clinical signs were recorded. Physical examination, thoracic radiography, and electrocardiography were evaluated. Dogs with infectious, systemic, or other cardiovascular diseases that may cause pulmonary hypertension were excluded, as were those with a positive result in heartworm antigen test. The dogs were then divided into three groups including the normal, DMVD, and DMVD with PH (DMVD+PH) groups based on radiographic and echocardiographic findings.

### Echocardiography

Echocardiography was performed by an experienced cardiologist using an ultrasound machine (M9, Mindray, China) with a 4–12 MHz phased array transducer to diagnose DMVD. Dogs with normal cardiac structure and function by echocardiography were categorized in the normal group.

The DMVD group were those diagnosed as DMVD stage C by radiographic signs of pulmonary oedema suggesting left-sided congestive heart failure, and echocardiographic findings including mitral valve thickening, mitral regurgitation, and left atrial and left ventricular enlargement. The enlargement of left atrium and ventricle was identified by a left atrium to aorta (LA/Ao) ratio of >1.6 in the right parasternal short-axis view in early diastole, and a normalized left ventricular internal diameter at end-diastole (LVIDd) of >1.7 cm/kg (22).

The DMVD+PH group consisted of DMVD stage C dogs with intermediate to high probability PH. The intermediate probability of PH was characterized by peak tricuspid regurgitation (TR) velocity of > 3 m/s with or without one anatomic site of echocardiographic signs of PH. The high probability of PH was characterized by a peak (TR) velocity of >3 m/s with >2 anatomic sites of echocardiographic signs of PH, or a peak (TR) velocity of >3.4 m/s with >1 anatomic sites of echocardiographic signs of PH (1). The anatomic sites of echocardiographic signs of PH included (1) ventricles [interventricular septal flattening, underfilling or decreased size of left ventricle, right ventricular hypertrophy or right ventricular systolic dysfunction], (2) pulmonary artery [pulmonary artery enlargement (PA/Ao > 1.0), peak early diastolic pulmonary regurgitation velocity >2.5 m/s, right pulmonary artery distensibility index <30%, right ventricular outflow Doppler acceleration time (<52–58 ms), acceleration time to ejection time ratio (<0.30) or systolic notching of the Doppler right ventricle outflow profile], and (3) right atrium and caudal vena cava [right atrial enlargement or enlargement of caudal vena cava]. Dogs were subdivided into two groups of intermediate and high probability of PH for sub-analysis.

### Sample Collection and Preparation

All dogs were fasted for at least 8 h before blood collection. Those fed with diets containing substantial levels of serotonin, such as bananas, kiwi fruits, plums, pineapple, and tomatoes, were excluded (23). Four milliliters (ml) of blood were collected by venipuncture from the cephalic vein with a 21-gauge needle and transferred to 5-ml evacuated tube containing EDTA as an anticoagulant. All samples were then processed within 2 h after collection. One milliliter of each sample was used for the analysis of routine hematology and blood chemistry profiles. The number of platelets was counted manually and the mean platelet volume (MPV) noted.

The remaining 3 ml of blood was used for measuring platelet and plasma serotonin concentrations. The blood samples were processed for preparing platelet-rich plasma (PRP) and platelet-poor plasma (PPP) samples, following the manufacturers' instructions (IBL International GMBH, Germany). In brief, the whole blood was centrifuged at 200 g for 10 min and the supernatant, the PRP, was collected. To measure the platelet serotonin concentration, 200 μl of PRP was transferred and added to 800 μl of physiological saline for measurement of platelet serotonin concentration. The diluted PRP was then centrifuged at 4,500 g for 10 min at 4°C and the supernatant discarded. The pellets were added to 200 μl of distilled water and stored at −20°C until assay, when the samples were thawed, centrifuged at 10,000 g for 2 min and the supernatant aspirated for measurement of platelet serotonin concentration. The remaining PRP was centrifuged at 1,500 g for 20 min and collected the supernatant collected as the PPP for measurement of plasma serotonin concentration.

### Platelet and Plasma Serotonin Concentration Measurement

The prepared samples were measured for platelet and plasma serotonin concentrations using the competitive ELISA test kits: the serotonin ELISA (IBL International GMBH, Cat# RE59121, Germany) test and the serotonin high-sensitivity ELISA test (IBL International GMBH, Cat# RE59141, Germany), respectively. The optical density of the reactions of platelet and plasma samples were measured with a photometer (Epoch 2 microplate reader, BioTek, USA) at 405 and 450 nm, respectively. The in-house ELISA validation was performed for intra- and inter-assay precision. Five spiked samples with different concentrations were used for spike-recovery assessment.

### Statistical Analysis

Statistical analysis was performed by the computer-based software, SPSS version 22 (IBM, USA). The differences in serotonin concentrations among the three groups were analyzed by the Kruskal–Wallis test and Dunn's *post-hoc* test, and comparisons between the two groups were made by the Mann–Whitney *U*-test. The data were presented in the median and interquartile range (IQR). The correlations between circulating serotonin concentration and age, body weight, platelet count, MPV, and PAP were analyzed by the Spearman correlation and multivariable regression analysis was used to analyse the association between plasma or platelet serotonin concentrations and sex and breed of the dogs. A significant difference was accepted at *p* <0.05.

## Results

Sixty-two dogs were enrolled in the present study and divided into three groups including the normal (*n* = 22), DMVD (*n* = 20), and DMVD+PH (*n* = 20) groups. The normal group consisted of eight males (one intact and seven castrated males) and 14 sterilized females with eight Shih Tzus, five Chihuahuas, five Yorkshire Terriers, one Dachshund, and three mixed breed dogs. The DMVD group was composed of 15 males (nine intact and six castrated males) and five sterilized females with five Chihuahuas, five Pomeranians, four Poodles, one Yorkshire Terrier, one Shih Tzu, one Chinese Crested Hairless Dog, one Finnish Spitz, one Beagle, and one mixed breed dog. The DMVD+PH group comprised nine males (four intact and five castrated males) and 11 females (three intact and eight sterilized females) with six Poodles, five Chihuahuas, two Miniature Pinschers, two Shih Tzus, one Jack Russel Terrier, and four mixed breed dogs. The ages of dogs in the normal group were significantly younger than those of DMVD and DMVD+PH groups (Table 1). Breeds and sex were not different among groups. The clinical signs of the DMVD and DMVD+PH groups included coughing (16/20 and 17/20), exercise intolerance (9/20 and 9/20), respiratory distress (4/20 and 6/20), and syncope (1/20 and 3/20). The physical examination of the DMVD and DMVD+PH groups was systolic murmur (20/20 and 20/20), increased lung sound (9/20 and 15/20), lung crackles (4/20 and 4/20), pale pink mucous membrane (1/20 and 5/20), and ascites (0/20 and 4/20). The Chi-square test revealed no difference of the clinical signs from history taking and physical examination between male and female dogs (*p* > 0.05). The clinical appearances are summarized in Table 1. The hematology and blood chemistry profiles including platelet counts and MPV were within the normal ranges. There was no significant difference in hematology and blood chemistry profiles among groups except a greater blood urea nitrogen (BUN) in the DMVD and DMVD+PH groups when compared to the normal group (*p* < 0.05). The echocardiographic findings showed that LA/Ao, LVIDd and %fractional shortening (%FS) of dogs in the DMVD and DMVD+PH groups were greater than the normal group (*p* < 0.05) and left ventricular internal diameter at end-systole (LVIDs) of dogs in the DMVD group were larger than the normal group (*p* < 0.05; Table 1). DMVD dogs with an intermediate probability of PH (*n* = 11) had a lower peak TR velocity and estimated PAP than those with a high probability of PH (*n* = 9; *p* = 0.004; Table 2).

**Table 1:** Summary of signalment, echocardiographic data, serotonin concentrations, platelet counts, and mean platelet volume of normal dogs and degenerative mitral valve disease dogs (DMVD) and DMVD dogs with pulmonary hypertension (PH).

	**Normal (*n* = 22)**	**DMVD (*n* = 20)**	**DMVD+PH (*n* = 20)**	*p*-value^*^
Gender (male/female)	8/14	15/5	9/11	–
Age (years)	8.00 (7.00–9.75)	12.00 (11.00–13.25)^a^	12.00 (10.00–14.00)^a^	<0.0001
Weight (kg)	4.65 (2.87–6.00)	5.16 (3.95–6.51)	5.22 (4.47–6.16)	0.191
LA index (cm/kg)	1.00 (0.93–1.08)	2.23 (1.91–2.59)^a^	1.69 (1.40–2.50)^a^	0.000
Ao index (cm/kg)	0.78 (0.69–0.88)	1.18 (0.94–1.28)^a^, ^b^	0.81 (0.67–1.13)^b^	0.007
LA/Ao	1.27 (1.56–1.40)	1.82 (1.58–2.34)^a^	2.19 (2.06–2.24)^a^	<0.0001
IVSd index (cm/kg)	0.45 (0.40–0.48)	0.47 (0.40–0.50)	0.45 (0.40–0.54)	0.725
LVIDd index (cm/kg)	1.27 (1.21–1.39)	1.86 (1.73–1.96)^a^	1.80 (1.75–1.98)^a^	0.000
LVPWd index (cm/kg)	0.37 (0.33–0.42)	0.37 (0.34–0.43)	0.40 (0.35–0.43)	0.886
IVSs index (cm/kg)	0.59 (0.51–0.63)	0.57 (0.52–0.64)	0.63 (0.57–0.73)^a^	0.137
LVIDs index (cm/kg)	0.75 (0.72–0.88)	1.02 (0.87–1.74)^a^	0.77 (0.68–1.09)	0.009
LVPWs index (cm/kg)	0.64 (0.56–0.68)	0.66 (0.61–0.74)	0.70(0.58–0.79)	0.226
%FS	38.52 (35.53–43.78)	44.15 (38.47–47.61)^a^	50.65 (43.34–56.37)^a^	0.001
PAP (mmHg)	–	–	52.88 (44.66–67.16)	–
Platelet serotonin (ng/10^9^ platelets)	179.73 (102.37–352.24)	325.99 (96.84–407.66)^c^	135.11 (21.21–312.22)^c^	0.081
Plasma serotonin (ng/ml)	2.92 (1.76–7.50)	1.23 (0.27–4.23)	1.75 (1.19–2.72)	0.920
Platelet counts (× 10^3^ cells/ml)	268.50 (223.00–323.75)	303.00 (252.50–400.50)	299.50 (245.50–367.25)	0.267
MPV (fL)	10.00 (8.90–10.50)	9.50 (9.10–11.05)	10.20 (9.55–11.25)	0.621

*Ao, aorta; FS, fractional shortening; IVSd, interventricular septal thickness at end diastole; IVSd, interventricular septal thickness at end systole; LA, left atrium; LA/Ao, left atrium to aorta ratio; LVIDd, left ventricular internal diameter at end diastole; LVIDs, left ventricular internal diameter at end systole; LVPWd, left ventricular posterior wall thickness at end diastole; LVPWs, left ventricular posterior wall thickness at end systole; MPV, mean platelet volume; PAP, pulmonary arterial pressure*.

*The data are expressed as median (interquartile range)*.

* *The p-values represent the significant difference among three groups by the Kruskal–Wallis test*.

a *Significant difference compared to the normal group at p < 0.05*.

b *Significant difference between the DMVD and the DMVD+PH group at p < 0.05*.

c *Significant difference between DMVD and DMVD+PH group at p < 0.05 analyzed by the Mann–Whitney U-test*.

**Table 2:** Platelet and plasma serotonin concentration of dogs in normal, degenerative mitral valve disease (DMVD), DMVD with intermediate probability of pulmonary hypertension (PH) and DMVD with high probability of PH groups, and peak tricuspid regurgitation (TR) velocity and estimated pulmonary arterial pressure (PAP) of DMVD with intermediate probability of PH and DMVD with high probability of PH group.

	**Normal (*n* = 22)**	**DMVD (*n* = 20)**	**DMVD with intermediate probability of PH (*n* = 11)**	**DMVD with high probability of PH (*n* = 9)**	***p*-value**
Platelet serotonin concentration (ng/10^9^ platelets)	179.73 (102.37–352.24)	325.99 (96.84–407.66)^a^	291.11 (106.69–400.84)	35.82 (2.69–126.35)^a^	0.014
Plasma serotonin concentration (ng/ml)	2.92 (1.76–7.50)	1.23 (0.27–4.23)	1.50 (1.22–1.93)	2.37 (0.57–3.49)	0.175
Peak TR velocity (m/s)	–	–	3.40 (3.29–3.62)	4.12 (3.76–4.59)	0.004
PAP (mmHg)	–	–	46.19 (43.42–52.43)	67.90 (56.55–84.31)	0.004

*The p-values represent the significant difference among groups. The platelet and plasma serotonin concentrations were analyzed by the Kruskal–Wallis test. The peak TR velocity and PAP were analyzed by Mann–Whitney U-test*.

a *Significant difference between DMVD and DMVD with high probability of PH groups analyzed by Dunn's post-hoc test at p < 0.05*.

*Data are presented as median (interquartile range)*.

### Platelet and Plasma Serotonin Concentrations

**Figure 1:**
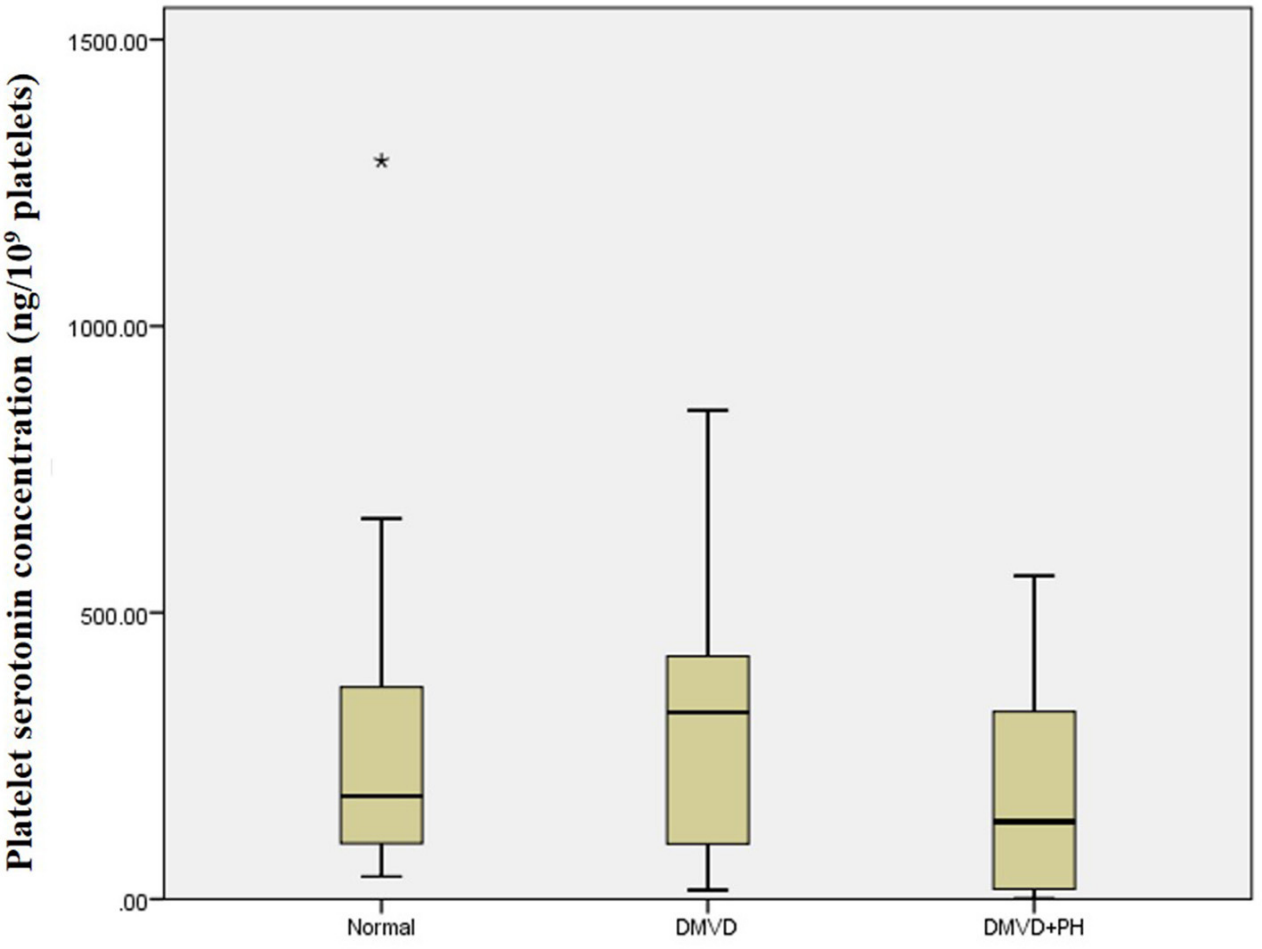
Box plot of platelet serotonin concentration of the normal (*n* = 22), degenerative mitral valve disease (DMVD) (*n* = 20) and degenerative mitral valve disease and pulmonary hypertension (DMVD+PH) (*n* = 20) groups. Median values of platelet serotonin concentration are shown as horizontal lines. The ends of the box are the 25th and 75th quartiles. The ends of the whiskers represent the minimum and maximum of all the data. Data not included between the whiskers are plotted as an outlier with an asterisk.

**Figure 2:**
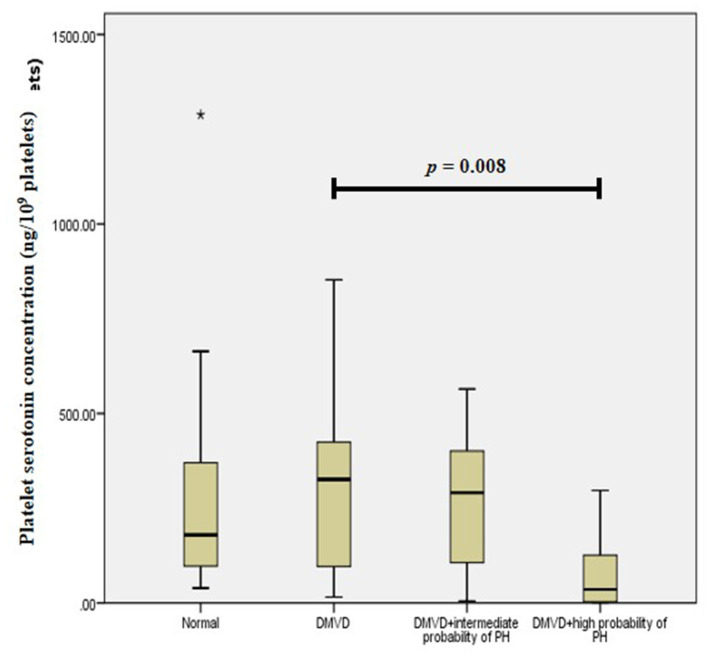
Box plot of platelet serotonin concentration of the normal (*n* = 22), degenerative mitral valve disease (DMVD) (*n* = 20), degenerative mitral valve disease with intermediate probability of pulmonary hypertension (DMVD+intermediate probability of PH) (*n* = 11) and degenerative mitral valve disease with high probability of pulmonary hypertension (DMVD+high probability of PH) (*n* = 9). There was the significant difference between groups (*p* = 0.014). The platelet serotonin concentrations of DMVD dogs without PH were significantly higher than those of DMVD dogs with high probability of PH (*p* = 0.008). Median values of platelet serotonin concentration are shown as horizontal lines. The ends of the box are the 25th and 75th quartiles. The ends of the whiskers represent the minimum and maximum of all the data. Data not included between the whiskers are plotted as an outlier with an asterisk (*).

The Kruskal–Wallis test showed no difference in the platelet and plasma serotonin concentrations among the three groups (Table 1 and Figure 1). However, comparison of platelet serotonin concentration between the four groups of normal (179.73 [102.37–352.24] ng/10^9^ platelets), DMVD (325.99 [96.84–407.66] ng/10^9^ platelets), DMVD with intermediate probability of PH (291.11 [106.69–400.84] ng/10^9^ platelets) and DMVD with high probability of PH (35.82 [2.69–126.35] ng/10^9^ platelets) exhibited the significant difference (*p* = 0.014). The Dunn's *post-hoc* test revealed that the platelet serotonin concentration of DMVD dogs with high probability of PH was lower than those in the DMVD group (*p* = 0.008; Table 2 and Figure 2). However, there was no significantly different between other groups.

The medians of platelet and plasma serotonin concentrations are shown in Table 1. Platelet and plasma serotonin concentration between male and female dogs was not significant difference (194.99 [96.08–373.64] ng/10^9^ platelets vs. 226.37 [80.29–405.16] ng/10^9^ platelets, *p* = 0.789 and 2.14 [1.14–4.53] vs. 1.89 [0.57–4.90], *p* = 0.828, respectively).

**Figure 3:**
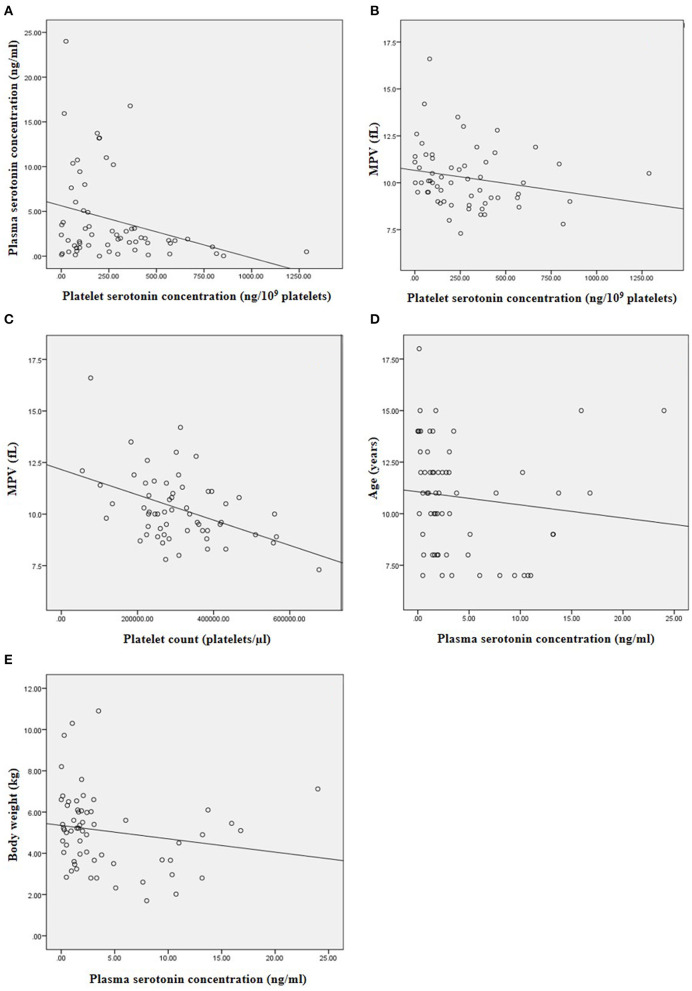
The correlation between the variables in this study. **(A)** Negative correlation between platelet and plasma serotonin concentration (*r* = −0.253, *p* = 0.049). **(B)** Negative correlation between platelet serotonin concentration and MPV (*r* = −0.297, *p* = 0.022). **(C)** Negative correlation between platelet count and MPV (*r* = −0.345, *p* = 0.007). **(D)** Negative correlation between plasma serotonin concentration and age of the dogs (*r* = −0.353, *p* = 0.005). **(E)** Negative correlation between plasma serotonin concentration and body weight of the dogs (*r* = −0.281, *p* = 0.028).

There was negative correlation between platelet and plasma serotonin concentrations (*r* = −0.253, *p* = 0.049) (Figure 3A). Platelet serotonin concentration and platelet count were negatively correlated with MPV (*r* = −0.297, *p* = 0.022 and *r* = −0.345, *p* = 0.007, respectively; Figures 3B,C). There was no association between plasma and platelet serotonin concentrations and sex and breed of dogs. Age and body weight were negatively correlated with plasma serotonin concentration (*r* = −0.353, *p* = 0.005 and *r* = −0.281, *p* = 0.028, respectively; Figures 3D,E). The estimated PAP did not correlate with plasma and platelet serotonin concentrations in the DMVD+PH groups.

In-house ELISA validation tests of the serotonin ELISA and the serotonin high sensitive ELISA tests showed intra-assay precision (6.56 and 4.89%, respectively), inter-assay precision (6.54 and 9.16%, respectively), and spike-recovery assessment (104.19 and 101.00%, respectively).

## Discussion

The study investigated the differences in platelet and plasma serotonin concentrations among normal and DMVD dogs with or without PH. The main finding of this study was a decrease in platelet serotonin concentration in DMVD dogs with a high probability of PH compared to those DMVD dogs without PH. The plasma serotonin concentration was not different between normal and DMVD dogs with or without PH.

The results of the study showed no difference in the platelet serotonin concentration between normal and DMVD dogs; however, there was a trend of increasing platelet serotonin concentration in DMVD dogs. This finding is in agreement with a previous study in small-breed dogs aged more than 6 years old and weight <10 kg with 10 males and 10 females in normal group and 11 males and 12 females in DMVD group (24). On the other hand, another study between the normal large-breed dogs aged more than 3 years old and weight more than 20 kg (16 males and 20 females), and the small-breed DMVD dogs with weight <15 kg [26 males and 24 females with 37 Cavalier King Charles spaniel dogs (CKCS) and 13 non-CKCS dogs] showed a higher serum serotonin concentration in the DMVD dogs than in normal dogs (25). Normally, serotonin is stored in dense granules in the cytosol of platelets (14, 26), and serum serotonin concentration seems to be an approximate sum of platelet and plasma serotonin concentrations (27). Therefore, plasma serotonin may be a better measure for evaluating serotonin in circulation in conditions without platelet activation. A previous study reported an increase in serotonin release from platelets during *in vitro* platelet aggregation, thus decreasing platelet serotonin concentration and increasing the plasma serotonin concentration (8). This finding is similar to the results of the present study, which found a negative correlation between platelet and plasma serotonin concentrations. An increase in plasma and serum serotonin in DMVD dogs suggests a relationship between circulating serotonin and DMVD; however, whether this is the cause or effect of DMVD has not yet been proved.

The difference in breed can affect the serum serotonin levels in healthy dogs. For example, the CKCS has a higher serum serotonin concentration than other small- breed dogs (28). Moreover, the serum and plasma serotonin concentrations of CKCS dogs with mild DMVD was higher than those of dogs with severe DMVD and congestive heart failure (29). Therefore, this research was designed as a breed-matched study. The result showed no correlation between breed and plasma or platelet serotonin concentrations suggesting a minimal interbreed variation of platelet and plasma serotonin concentrations.

Sex is another factor that has effects on serum serotonin concentration. A previous study showed that female dogs had higher serum serotonin concentrations than males (28). In humans, women have a higher incidence of PH than men (30). It has been suggested that estrogen is involved in the regulation of the mediators in the serotonin signaling pathway in animal models and in human patients affected with pulmonary arterial hypertension (30). The present study did not provide sex-matched dogs among groups; the majority of dogs in the DMVD group were males. However, male and female dogs in this study had similar plasma and platelet serotonin concentrations. In addition, sex did not correlate with plasma or platelet serotonin concentrations. Therefore, a profound effect of sex on serotonin concentrations was excluded.

The age of dogs in the control group was lower than that in the DMVD and DMVD+PH groups. Also, age was negatively correlated with plasma serotonin concentration. A similar result has been observed in a previous study that found a negative correlation between age and serum serotonin concentration. These findings suggest a profound effect of a decrease in circulating serotonin concentrations in older dogs (25).

A previous study showed a negative correlation between body weight and plasma serotonin (31); however, the body weights of dogs in all three groups in the present study were matched. The effect of body weight on serotonin concentration was therefore reduced.

The plasma serotonin concentrations of normal and DMVD+PH dogs were not different. This result was similar to that of a previous study on human patients with pulmonary arterial hypertension and PH secondary to chronic thromboembolism compared to controls (32). We also did not find a difference in plasma serotonin concentration between the DMVD and DMVD+PH groups. However, a decrease in platelet serotonin concentration was found in the DMVD dogs with high probability of PH compared to the DMVD dogs without PH, suggesting a significant drop of platelet serotonin concentration only in DMVD dogs with a high degree of PH. The concentration of platelet serotonin in DMVD dogs with an intermediate probability of PH was still similar to those without PH. It has been suggested that platelet serotonin concentration may decrease following an increased serotonin release from platelets to pulmonary artery endothelial and smooth muscle cells in human patients with PH (32). Therefore, based on the result of this study, it is speculated that the massive use of platelet serotonin may occur mainly in the advanced stage of PH.

MPV reflects platelet production (33, 34), and a decrease in MPV indicates platelets of small size, reflecting an excessive platelet production. On the other hand, an increase in MPV indicates a large platelet size, manifesting an inadequate platelet production. This suggestion was supported by the result of the present study, which showed a negative correlation between MPV and platelet count and platelet serotonin concentration. A previous study on human patients with PH secondary to chronic obstructive pulmonary disease (COPD) showed a higher MPV in human patients with PH than those without PH. In addition, MPV was positively correlated with PAP, suggesting an association between MPV and PH severity in human patients (35). However, the present study failed to find a difference in MVP among groups, suggesting a similar platelet production in normal dogs and DMVD dogs with or without PH.

The first limitation of the present study was a small sample size, which may affect the significance of the data; further studies with a larger number of samples should be performed. Another limitation of this study was the duration of sample collection. All blood sample collection could not be operated simultaneously; however, care was taken to follow the manufactures' recommendations and prevent serotonin degradation by storing blood samples for no longer than a year at −80°C and avoiding repeated freeze–thaw cycles.

In conclusion, the study showed a decrease in platelet serotonin concentration in DMVD dogs with PH associated with the degree of PH probability. However, the plasma serotonin concentration was not different between normal and DMVD dogs with or without PH. Further investigations should be performed to clarify the role of platelet serotonin and PH secondary to DMVD in dogs.

## Data Availability Statement

The raw data supporting the conclusions of this article will be made available by the authors, without undue reservation.

## Ethics Statement

The animal study was reviewed and approved by the Institutional Animal Care and Use Committee, Faculty of Veterinary Science, Chulalongkorn University (number 33053). Written informed consent was obtained from the owners for the participation of their animals in this study.

## Author Contributions

NT was responsible for sample collection, laboratory work, data analysis, and manuscript writing and revision. PM was responsible for laboratory work. SS was responsible for data analysis, and manuscript revision and editing. All authors contributed to the article and approved the submitted version.

## Conflict of Interest

The authors declare that the research was conducted in the absence of any commercial or financial relationships that could be construed as a potential conflict of interest.

## Abbreviations

Ao: aorta
CKCS: Cavalier King Charles Spaniel
DMVD: degenerative mitral valve disease
ERK $\frac{1}{2}$: extracellular signal-regulated kinase $\frac{1}{2}$
%FS: %fractional shortening
IQR: interquartile range
LA: Left atrium
LVIDd: left ventricular internal diameter at end-diastole
LVIDs: left ventricular internal diameter at end-systole
MPV: mean platelet volume
PAP: pulmonary arterial pressure
pERK1/2: phosphorylated extracellular signal-regulated kinase $\frac{1}{2}$
PH: pulmonary hypertension
PPP: platelet-poor plasma
PRP: platelet-rich plasma
SERT: serotonin transporter
TPH-1: tryptophan hydroxylase 1
TR: tricuspid regurgitation.
